# Bleomycin-induced flagellate-like skin pigmentation in a 15-year-old girl: a case report and review

**DOI:** 10.3389/fmed.2025.1610203

**Published:** 2025-08-05

**Authors:** Xinrong Chen, Chuangjie Zheng, Ke Wang, Linzhu Zhai

**Affiliations:** ^1^The First Clinical Medical College of Guangzhou University of Chinese Medicine, Guangzhou, Guangdong, China; ^2^Cancer Center, The First Affiliated Hospital of Guangzhou University of Chinese, Guangzhou, Guangdong, China

**Keywords:** bleomycin, flagellate hyperpigmentation, dermal toxicity, ovarian yolk sac tumor, adverse reactions

## Abstract

We present a detailed report of a case of flagellar-like skin pigmentation observed in a 15-year-old female patient diagnosed with ovarian yolk sac tumor following administration of a chemotherapy regimen containing bleomycin. After three cycles of chemotherapy, the patient exhibited characteristic painless, non-pruritic linear hyperpigmentation on her trunk and back. This case underscores the significance of paying attention to bleomycin-associated dermal toxicity, emphasizing the need for its early detection and management to alleviate the patient’s psychological and physical distress. Our research indicates that additional studies are necessary to comprehensively understand the underlying mechanisms of flagellar-like skin pigmentation and to identify effective prevention and treatment approaches.

## Introduction

Bleomycin, a chemotherapeutic agent, is widely used to treat a variety of malignant tumors, and it is particularly significant in the context of solid tumors, including ovarian tumors. Despite its antitumor properties, bleomycin administration can result in distinctive cutaneous side effects, with a reported incidence of flagellate-like skin pigmentation ranging from 8 to 20% (1). This side effect, although uncommon, can significantly impact the patient’s psychological well-being and aesthetic appearance. The pathogenesis of flagellar skin pigmentation remains incompletely understood, and information regarding its treatment and prognosis is relatively scarce (2). This article presents a case study of a 15-year-old female patient who developed flagellar-like skin pigmentation following bleomycin treatment, aiming to shed light on the clinical characteristics, underlying mechanisms, and approaches for the management of this side effect. In addition, we review relevant literature on bleomycin-induced flagellate hyperpigmentation to contextualize this adverse effect.

## Case report

**Figure 1:**
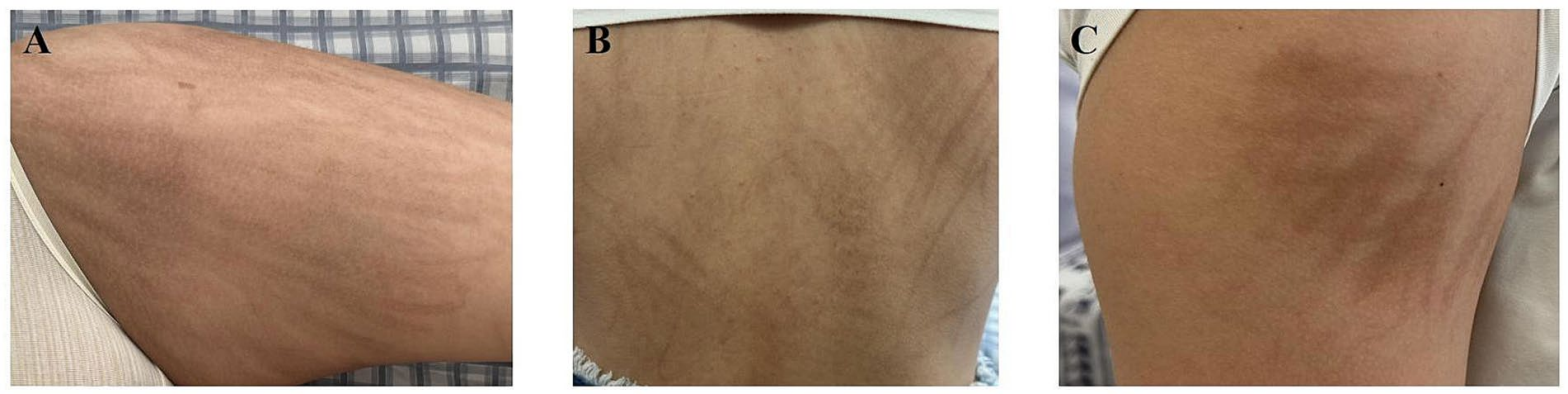
Clinical photographs of bleomycin-induced flagellate hyperpigmentation. **(A)** the thigh, **(B)** the trunk, and **(C)** the arm.

A 15-year-old girl presented with lower abdominal distension and pain without clear triggers. An abdominal CT scan showed a pelvic space-occupying lesion measuring approximately 108 × 185 × 212 mm. An open left salpingo-oophorectomy and resection of peritoneal carcinomatous foci were performed. The postoperative pathology indicated ovarian yolk sac tumor, with tumor tissue observed in the abdomen. The immunohistochemistry results were as follows: CK (+), SALL4 (+), CD117 (+), CD30 (−), AFP (++++), P53 (10%+), and Ki-67 (70%, +). Staging procedures were conducted, involving a bone biopsy, bone marrow aspiration, lumbar puncture, and a whole-body CT scan, none of which revealed new findings. The definitive diagnosis was an ovarian yolk sac tumor, classified as stage T2N1M0 IIIA1. Five months post-surgery, commencing from March 2023, the patient underwent six courses of combination chemotherapy, which included cisplatin (45 mg, days 1–3), etoposide (0.22 g, days 1–3), and bleomycin (20 mg, day 1). By the third cycle, the cumulative bleomycin dose was approximately 60 mg. Following the third chemotherapy cycle, she exhibited painless, non-pruritic linear skin pigmentation on her trunk and back, resembling flagellation (Figure 1). In addition, the patient experienced alopecia, but without alterations in nail or mucous membrane appearance, and she exhibited no dyspnea or pulmonary symptoms.

Subsequent PET-CT imaging, conducted for reevaluation, revealed minimal residual disease foci in the peritoneum, liver capsule, and pelvic lymph nodes. In response, the treatment regimen was revised to encompass six courses of combination chemotherapy, including albumin-bound paclitaxel, cisplatin, and ifosfamide, within 4 months. Following chemotherapy completion, laparoscopic resection of the intrahepatic lesion was conducted, revealing intraoperative pathology indicative of a yellow granuloma without tumor tissue. Subsequent examinations indicated the disappearance of the patient’s intra-abdominal lesions, with tumor-related indices showing no significant abnormalities.

**Figure 2:**
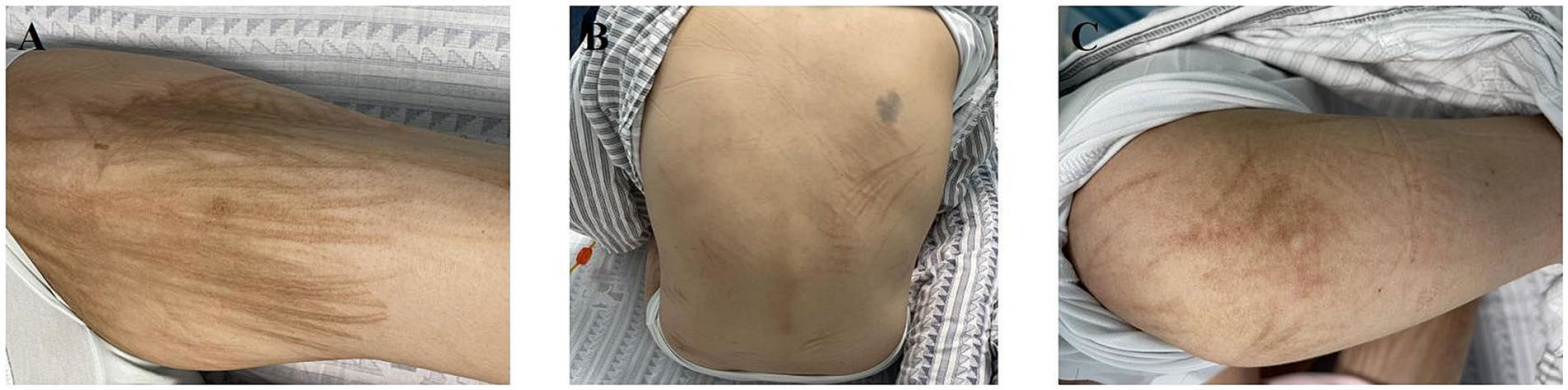
Clinical photographs of bleomycin-induced flagellar hyperpigmentation at 1-year follow-up on **(A)** the thigh, **(B)** the trunk, and **(C)** the arm.

At the 1-year follow-up after the onset of skin pigmentation, the patient continued to exhibit pigmentation but remained asymptomatic (Figure 2).

## Discussion

Hyperpigmentation involves either localized or widespread darkening of the skin or other tissues, which results from the deposition of pigment particles. This may be due to the accumulation of melanin, hemoglobin, or other pigmented substances in tissues. It is commonly linked to ultraviolet light exposure, hormonal fluctuations, inflammation, or adverse drug reactions (3).

Several case reports of bleomycin-induced flagellate hyperpigmentation have been documented, with occurrences following bleomycin treatment for conditions including Hodgkin’s lymphoma, reproductive system malignancies, and vascular malformations (4–9). We report a case of a 15-year-old girl diagnosed with an ovarian yolk sac tumor who exhibited flagellar-like skin pigmentation following chemotherapy treatment with bleomycin. Bleomycin, recognized for its antitumor properties, is extensively utilized in treating various cancers such as squamous cell carcinoma of the head and neck, skin cancer, testicular cancer, lymphoma, and malignant pleural effusion (10). However, the clinical relevance of its associated dermal toxicities, such as alopecia, eczema, erythema maculatum, sclerotic-like lesions, nail bed changes, Raynaud’s phenomenon, and hyperpigmentation, has become increasingly recognized. Clinical manifestations of flagellate-like skin pigmentation in patients receiving bleomycin typically present as scattered brown streaks resembling whip marks on the skin surface. This cutaneous adverse effect has been reported in approximately 8–20% of cases, with onset usually occurring between 1 and 9 weeks after initiation of treatment (11). These lesions are painless, non-pruritic, and may also present with punctate hemorrhages, pustules, and pus blisters. Flagellar-like skin pigmentation appears to be unrelated to the route of administration or the underlying malignancy. While it was previously believed to correlate with the cumulative dose of bleomycin, both existing literature and our current case indicate that the reaction may develop even during the initial treatment cycles. Thus, it should not be regarded as a strictly dose-dependent effect (12). Flagellate-like skin pigmentation has been reported following the administration of shiitake mushrooms (13), docetaxel infusion in metastatic breast cancer patients (14), and bendamustine infusion in individuals with refractory chronic lymphocytic leukemia (15).

The precise pathogenesis of bleomycin-induced flagellate pigmentation remains incompletely understood, but several mechanisms have been proposed. One hypothesis suggests that bleomycin stimulates local melanogenesis and induces inflammatory changes in the skin, possibly through neutrophil activation and increased exocrine sweat activity following intravenous administration (16). Another theory implicates direct cytotoxic effects of bleomycin on keratinocytes, leading to a fixed drug eruption localized to the skin. Importantly, this reaction is not considered an acute IgE-mediated (anaphylactic) hypersensitivity response but rather a delayed-type drug eruption or localized toxic effect confined to the cutaneous tissue (11). Clinical observations also indicate that pruritus frequently precedes the appearance of erythema and pigmentation. Scratching in response to this prodromal itching may facilitate local drug extravasation or concentration, contributing to the characteristic linear pattern of lesions (17).

Flagellate-like skin hyperpigmentation is not considered a severe adverse event and is typically self-limiting. Management is primarily supportive and may include prompt administration of antihistamines to relieve pruritus, topical or systemic corticosteroids to reduce inflammation, and non-steroidal anti-inflammatory drugs (NSAIDs) for symptomatic relief. In most cases, bleomycin therapy can be safely continued under close clinical monitoring. Discontinuation or dose reduction is generally recommended only when the cutaneous toxicity is particularly severe. Although permanent pigmentation is uncommon, the discoloration may persist for several weeks to months following cessation of bleomycin. Therefore, clinicians should inform patients of the potential for noticeable skin hyperpigmentation prior to treatment initiation, particularly due to its psychosocial impact. In the present case, bleomycin was initially administered alongside cisplatin and etoposide, and later replaced by a regimen containing albumin-bound paclitaxel, cisplatin, and ifosfamide. The patient’s pigmentation persisted beyond the discontinuation of bleomycin, raising the possibility that other chemotherapeutic agents may have contributed to the duration or intensity of the skin changes. However, the precise role of each agent in the development or persistence of flagellate pigmentation remains unclear, representing a limitation of this report. Further studies are needed to delineate the specific contributions of various chemotherapeutic drugs to this cutaneous reaction.

## Conclusion

Bleomycin-induced flagellate-like skin pigmentation is an uncommon but visually distinctive cutaneous adverse effect. Although not medically severe, its appearance can cause considerable psychological distress and should be recognized early during chemotherapy. Most cases are self-limiting and can be managed conservatively without discontinuing treatment. However, clinicians should remain vigilant for more pronounced cutaneous reactions, in which dose adjustment or cessation of bleomycin may be necessary. The case presented here highlights that this reaction may occur even at moderate cumulative doses and early in treatment, and that it can persist long after drug discontinuation. Clinicians should inform patients of this potential side effect prior to initiating therapy and monitor for early dermatologic signs.

## Data Availability

The original contributions presented in the study are included in the article/supplementary material, further inquiries can be directed to the corresponding author.
